# Supplementary material for: Short- vs. Long-Course Antibiotic Treatment for Acute Streptococcal Pharyngitis: Systematic Review and Meta-Analysis of Randomized Controlled Trials
Source: Antibiotics (Basel). 2020 Oct 26;9(11):733. doi: 10.3390/antibiotics9110733 (PMC7692631; doi:10.3390/antibiotics9110733)
Supplement: Supplementary file 1 [file antibiotics-09-00733-s001.pdf]

Supplemental Data

Short- versus long-course antibiotic treatment for acute streptococcal pharyngitis: systematic review and meta-analysis of randomised controlled trials.

Anna Engell Holm, Carl Llor, Lars Bjerrum, Gloria Cordoba

Content:

Supplemental Figure S1……………………………………………………………………page 2

Supplemental Figure S2……………………………………………………………………page 3

Supplemental Figure S3……………………………………………………………………page 4

Supplemental Figure S4……………………………………………………………………page 5

Supplemental Figure S5……………………………………………………………………page 6

Supplemental Figure S6……………………………………………………………………page 7

Supplemental Figure S7……………………………………………………………………page 8

Supplemental Figure S8……………………………………………………………………page 9

Supplemental Figure S9…………..………………………………………………………page 10

Study or Subgroup	Short-term penicillins Events	Short-term penicillins Total	Long-term penicillins Events	Long-term penicillins Total	Weight	Odds Ratio M-H, Random, 95% CI	Year
Gerber 1987	67	73	93	99	21.4%	0.72 [0.22, 2.33]	1987
Zwart 2000	58	96	79	92	39.3%	0.25 [0.12, 0.51]	2000
Skoog Ståhlgren 2019	190	212	197	210	39.4%	0.57 [0.28, 1.16]	2019
**Total (95% CI)**		**381**		**401**	**100.0%**	**0.43 [0.23, 0.82]**	
Total events	315		369				

Heterogeneity: $Tau^2 = 0.14$; $Chi^2 = 3.50$, df = 2 (P = 0.17); $I^2 = 43\%$

Test for overall effect: Z = 2.57 (P = 0.01)

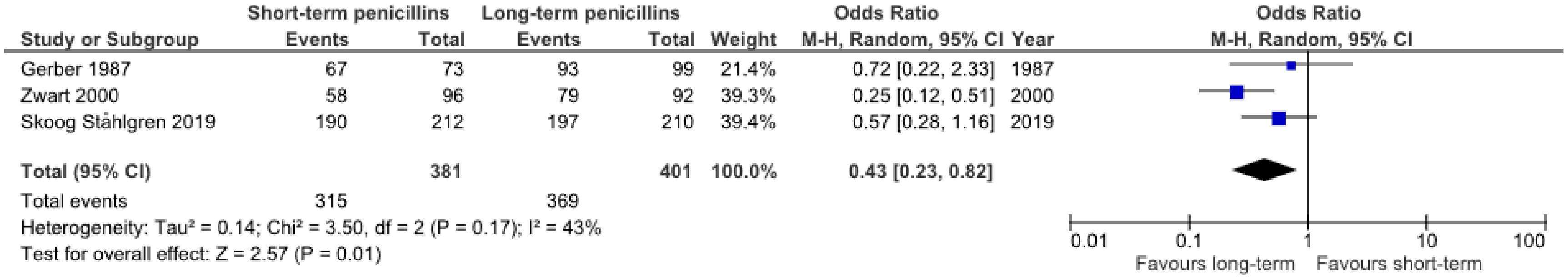

Fig. 1. Early clinical cure of short-term penicillins compared to long-term penicillin therapy

Figure S1. Early clinical cure of short-term penicillins compared to long-term penicillin therapy.

Study or Subgroup	Short-term macrolide Events	Total	Long-term penicillin Events	Total	Weight	Odds Ratio M-H, Random, 95% CI	Year
Hooton 1991	150	151	89	90	1.2%	1.69 [0.10, 27.28]	1991
Hamill 1993	40	41	41	41	0.9%	0.33 [0.01, 8.22]	1993
Portier 1995	123	130	128	132	4.7%	0.55 [0.16, 1.92]	1995
ODoherty 1996a	225	226	128	132	1.8%	7.03 [0.78, 63.59]	1996
Pacifico 1996	65	76	73	78	5.7%	0.40 [0.13, 1.23]	1996
Schaad 1996	149	160	143	160	8.9%	1.61 [0.73, 3.56]	1996
Adam 1996	100	102	97	99	2.2%	1.03 [0.14, 7.47]	1996
Gendrel 1997	98	102	106	108	2.8%	0.46 [0.08, 2.58]	1997
McCarty 2000	244	252	220	235	7.9%	2.08 [0.86, 5.00]	2000
Norrby 2001	110	138	119	150	12.4%	1.02 [0.58, 1.82]	2001
Portier 2001	103	114	97	109	8.0%	1.16 [0.49, 2.75]	2001
Portier 2002	156	168	159	165	6.6%	0.49 [0.18, 1.34]	2002
Schaad 2002	134	141	126	130	4.7%	0.61 [0.17, 2.13]	2002
Cohen 2002	292	334	155	167	10.7%	0.54 [0.28, 1.05]	2002
Takker 2003	174	177	145	154	4.3%	3.60 [0.96, 13.55]	2003
Norrby 2004	170	198	169	197	12.6%	1.01 [0.57, 1.77]	2004
Syrogiannopoulos 2004	253	267	132	135	4.7%	0.41 [0.12, 1.45]	2004
**Total (95% CI)**		**2777**		**2282**	**100.0%**	**0.93 [0.68, 1.26]**	
Total events	2586		2127				

Heterogeneity: $Tau^2 = 0.11$; $Chi^2 = 22.99$, df = 16 (P = 0.11); $I^2 = 30\%$
Test for overall effect: Z = 0.49 (P = 0.63)

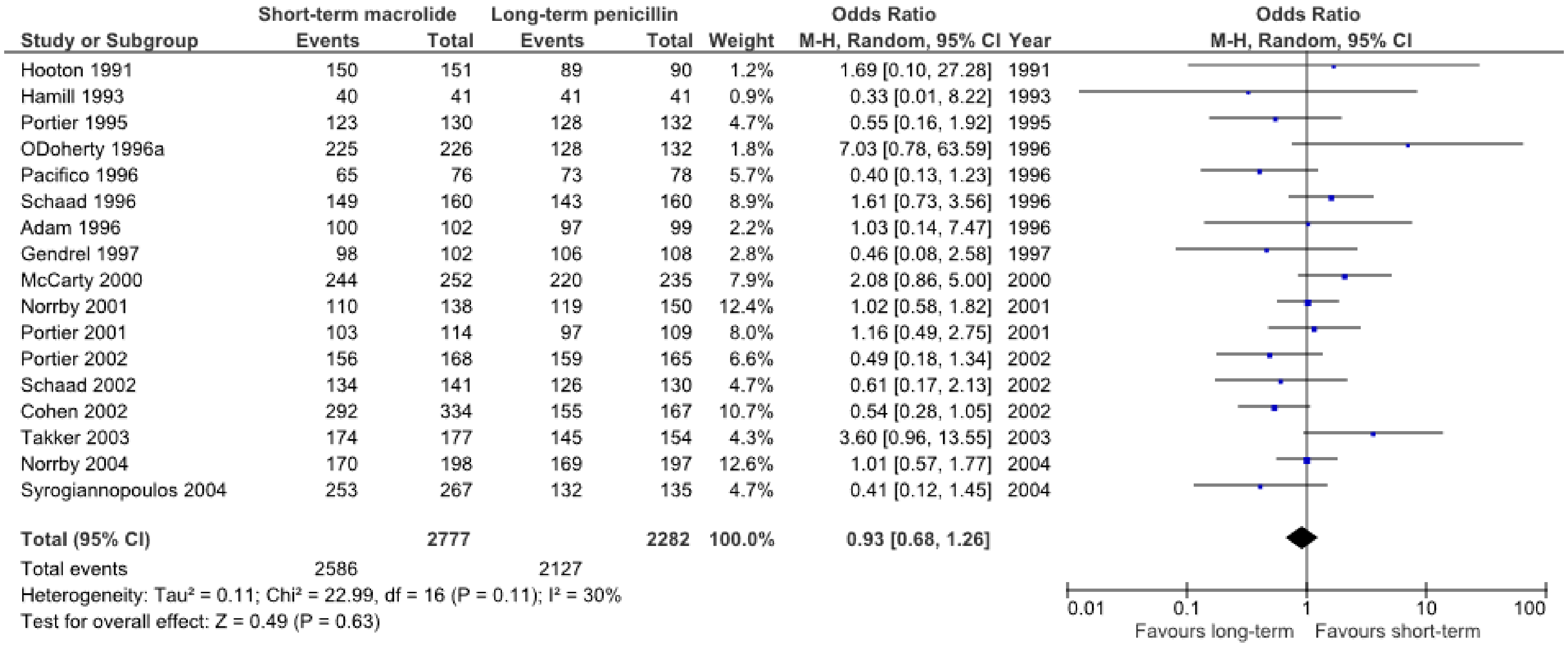

Fig. 2. Early clinical cure of short-term macrolides compared to long-term penicillin therapy

Figure S2. Early clinical cure of short-term macrolides compared to long-term penicillin therapy.

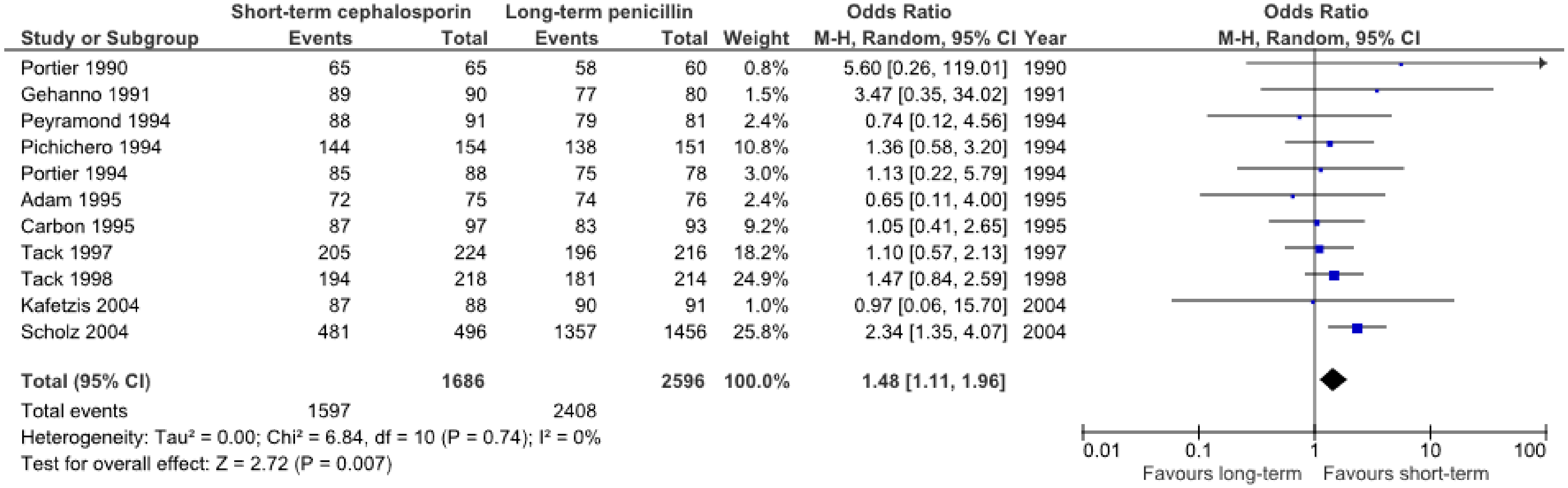

Study or Subgroup	Short-term cephalosporin Events	Short-term cephalosporin Total	Long-term penicillin Events	Long-term penicillin Total	Weight	Odds Ratio M-H, Random, 95% CI	Year
Portier 1990	65	65	58	60	0.8%	5.60 [0.26, 119.01]	1990
Gehanno 1991	89	90	77	80	1.5%	3.47 [0.35, 34.02]	1991
Peyramond 1994	88	91	79	81	2.4%	0.74 [0.12, 4.56]	1994
Pichichero 1994	144	154	138	151	10.8%	1.36 [0.58, 3.20]	1994
Portier 1994	85	88	75	78	3.0%	1.13 [0.22, 5.79]	1994
Adam 1995	72	75	74	76	2.4%	0.65 [0.11, 4.00]	1995
Carbon 1995	87	97	83	93	9.2%	1.05 [0.41, 2.65]	1995
Tack 1997	205	224	196	216	18.2%	1.10 [0.57, 2.13]	1997
Tack 1998	194	218	181	214	24.9%	1.47 [0.84, 2.59]	1998
Kafetzis 2004	87	88	90	91	1.0%	0.97 [0.06, 15.70]	2004
Scholz 2004	481	496	1357	1456	25.8%	2.34 [1.35, 4.07]	2004
**Total (95% CI)**		**1686**		**2596**	**100.0%**	**1.48 [1.11, 1.96]**	
Total events	1597		2408				

Heterogeneity: Tau² = 0.00; Chi² = 6.84, df = 10 (P = 0.74); I² = 0%
Test for overall effect: Z = 2.72 (P = 0.007)

Fig. 3. Early clinical cure of short-term cephalosporins compared to long-term penicillin therapy

Figure S3. Early clinical cure of short-term cephalosporins compared to long-term penicillin therapy.

Study or Subgroup	Short-term penicillin Events	Short-term penicillin Total	Long-term penicillin Events	Long-term penicillin Total	Weight	Odds Ratio M-H, Random, 95% CI	Year
Gerber 1987	67	73	92	99	16.9%	0.85 [0.27, 2.64]	1987
Stromberg 1988	47	67	128	136	22.5%	0.15 [0.06, 0.36]	1988
Zwart 2000	38	87	57	79	29.7%	0.30 [0.16, 0.57]	2000
Skoog Ståhlgren 2019	156	194	165	182	30.9%	0.42 [0.23, 0.78]	2019
**Total (95% CI)**		**421**		**496**	**100.0%**	**0.34 [0.19, 0.61]**	
Total events	308		442				

Heterogeneity: $Tau^2 = 0.19$; $Chi^2 = 6.59$, df = 3 (P = 0.09); $I^2 = 54\%$

Test for overall effect: Z = 3.65 (P = 0.0003)

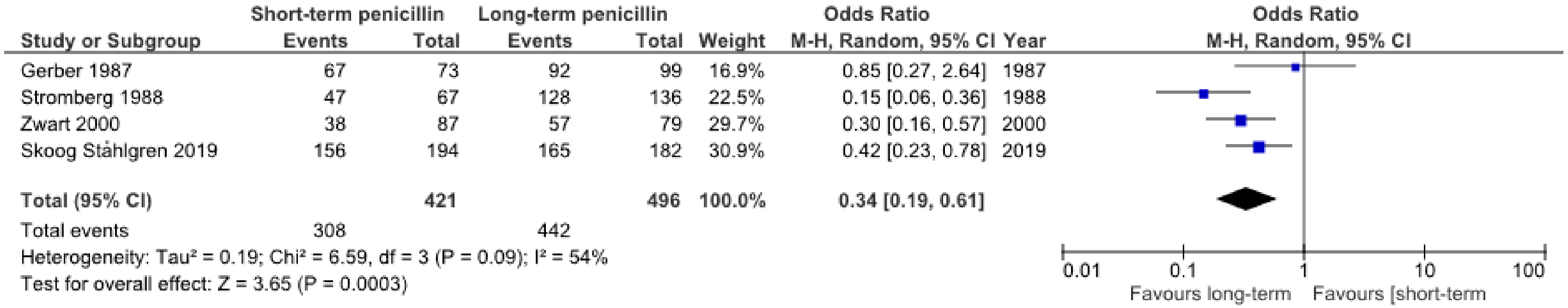

Fig. 4. Early bacteriological eradication of short-term penicillins compared to long-term penicillin therapy

Figure S4. Early bacteriological eradication of short-term penicillins compared to long-term penicillin therapy.

Study or Subgroup	Short-term macrolide Events	Short-term macrolide Total	Long-term penicillin Events	Long-term penicillin Total	Weight	Odds Ratio M-H, Random, 95% CI	Year
Hooton 1991	138	152	86	90	5.0%	0.46 [0.15, 1.44]	1991
Hamill 1993	39	41	42	44	3.0%	0.93 [0.12, 6.92]	1993
Portier 1995	93	98	89	101	5.1%	2.51 [0.85, 7.41]	1995
Adam 1996	85	102	87	99	5.9%	0.69 [0.31, 1.53]	1996
ODoherty 1996a	222	226	121	132	4.9%	5.05 [1.57, 16.19]	1996
Pacifico 1996	52	76	71	78	5.6%	0.21 [0.09, 0.53]	1996
Schaad 1996	99	152	128	156	6.6%	0.41 [0.24, 0.69]	1996
Gendrel 1997	81	102	97	108	5.9%	0.44 [0.20, 0.96]	1997
McCarty 2000	238	253	190	244	6.4%	4.51 [2.47, 8.24]	2000
Norrby 2001	111	138	119	150	6.5%	1.07 [0.60, 1.91]	2001
Portier 2001	93	113	87	109	6.2%	1.18 [0.60, 2.30]	2001
Cohen 2002	214	315	127	154	6.7%	0.45 [0.28, 0.73]	2002
Portier 2002	125	138	127	142	6.0%	1.14 [0.52, 2.48]	2002
Schaad 2002	54	141	105	130	6.5%	0.15 [0.09, 0.26]	2002
Takker 2003	158	192	140	168	6.5%	0.93 [0.54, 1.61]	2003
Syrogiannopoulos 2004	161	267	114	135	6.6%	0.28 [0.17, 0.47]	2004
Norrby 2004	110	138	119	150	6.5%	1.02 [0.58, 1.82]	2004
**Total (95% CI)**		**2644**		**2190**	**100.0%**	**0.76 [0.48, 1.20]**	
Total events	2073		1849				

Heterogeneity: Tau² = 0.77; Chi² = 119.09, df = 16 (P < 0.00001); I² = 87%
Test for overall effect: Z = 1.18 (P = 0.24)

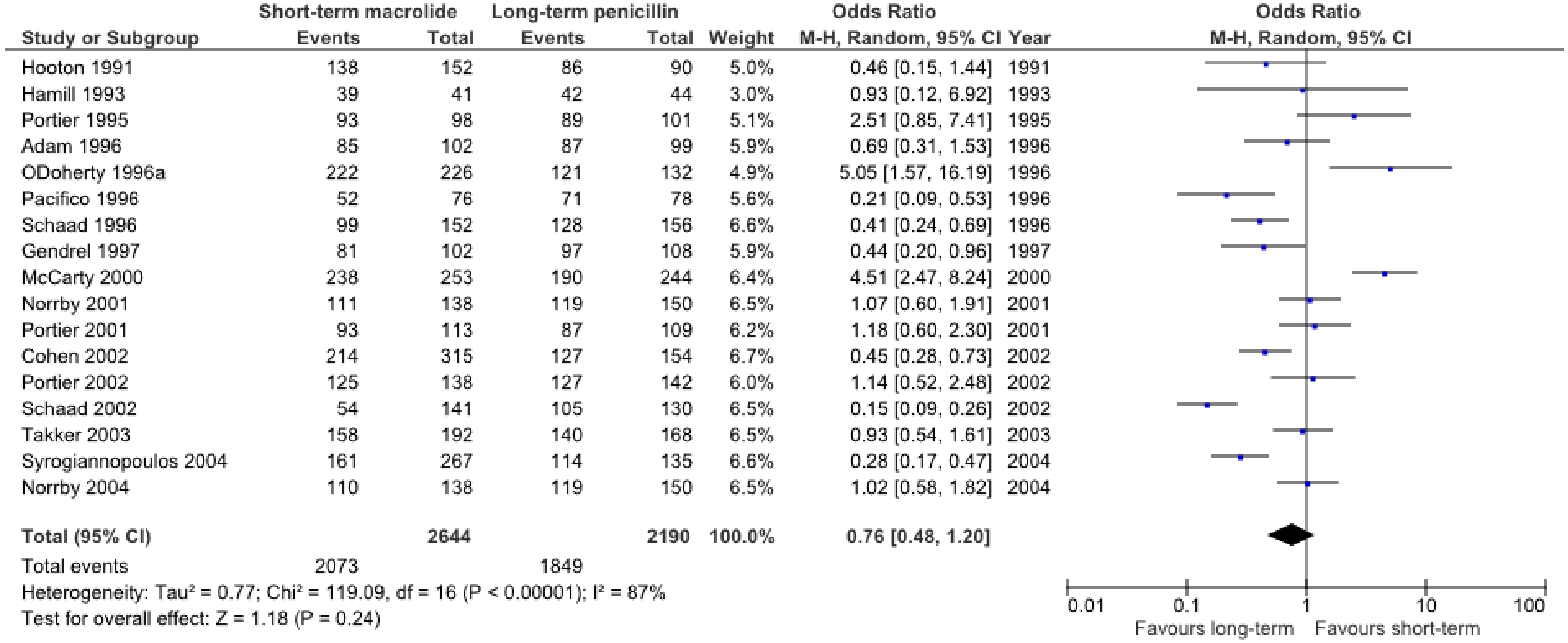

Fig. 5. Early bacteriological eradication of short-term macrolides compared to long-term penicillin therapy

Figure S5. Early bacteriological eradication of short-term macrolides compared to long-term penicillin therapy.

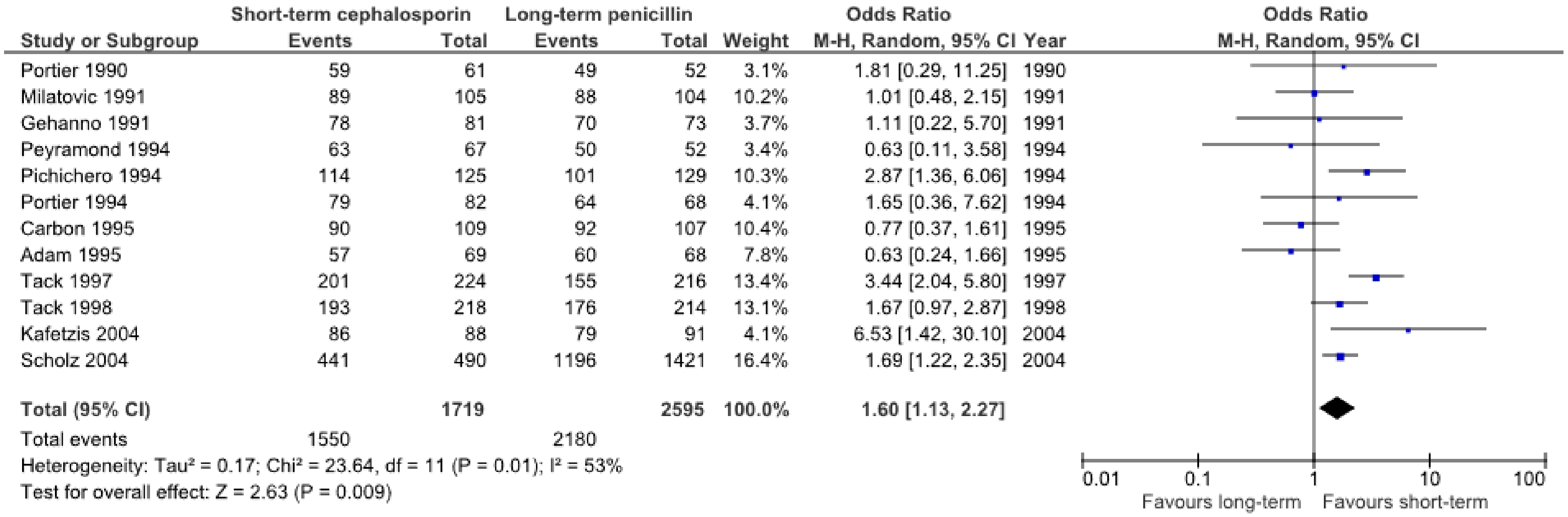

Study or Subgroup	Short-term cephalosporin Events	Short-term cephalosporin Total	Long-term penicillin Events	Long-term penicillin Total	Weight	Odds Ratio M-H, Random, 95% CI	Year
Portier 1990	59	61	49	52	3.1%	1.81 [0.29, 11.25]	1990
Milatovic 1991	89	105	88	104	10.2%	1.01 [0.48, 2.15]	1991
Gehanno 1991	78	81	70	73	3.7%	1.11 [0.22, 5.70]	1991
Peyramond 1994	63	67	50	52	3.4%	0.63 [0.11, 3.58]	1994
Pichichero 1994	114	125	101	129	10.3%	2.87 [1.36, 6.06]	1994
Portier 1994	79	82	64	68	4.1%	1.65 [0.36, 7.62]	1994
Carbon 1995	90	109	92	107	10.4%	0.77 [0.37, 1.61]	1995
Adam 1995	57	69	60	68	7.8%	0.63 [0.24, 1.66]	1995
Tack 1997	201	224	155	216	13.4%	3.44 [2.04, 5.80]	1997
Tack 1998	193	218	176	214	13.1%	1.67 [0.97, 2.87]	1998
Kafetzis 2004	86	88	79	91	4.1%	6.53 [1.42, 30.10]	2004
Scholz 2004	441	490	1196	1421	16.4%	1.69 [1.22, 2.35]	2004
**Total (95% CI)**		**1719**		**2595**	**100.0%**	**1.60 [1.13, 2.27]**	
Total events	1550		2180				

Heterogeneity: Tau² = 0.17; Chi² = 23.64, df = 11 (P = 0.01); I² = 53%
Test for overall effect: Z = 2.63 (P = 0.009)

Fig. 6. Early bacteriological eradication of short-term cephalosporins compared to long-term penicillin therapy

Figure S6. Early bacteriological eradication of short-term cephalosporins compared to long-term penicillin therapy.

Study or Subgroup	Short-term antibiotics Events	Short-term antibiotics Total	Long-term antibiotics Events	Long-term antibiotics Total	Weight	Odds Ratio M-H, Random, 95% CI	Year
Portier 1990	58	60	52	53	0.3%	0.56 [0.05, 6.33]	1990
Gehanno 1991	85	90	76	80	0.9%	0.89 [0.23, 3.45]	1991
Peyramond 1994	78	84	69	73	1.0%	0.75 [0.20, 2.78]	1994
Portier 1994	77	80	67	68	0.3%	0.38 [0.04, 3.77]	1994
Carbon 1995	112	115	108	115	0.9%	2.42 [0.61, 9.60]	1995
Adam 1995	69	75	68	76	1.4%	1.35 [0.45, 4.11]	1995
Portier 1995	109	117	120	125	1.3%	0.57 [0.18, 1.79]	1995
Carbon 1996	175	176	83	83	0.2%	0.70 [0.03, 17.38]	1996
ODoherty 1996a	189	199	106	108	0.7%	0.36 [0.08, 1.66]	1996
ODoherty 1996b	149	152	156	156	0.2%	0.14 [0.01, 2.66]	1996
Pacifico 1996	57	65	71	73	0.7%	0.20 [0.04, 0.98]	1996
Muller 1996	88	89	86	92	0.4%	6.14 [0.72, 52.07]	1996
Adam 1996	89	102	93	99	1.7%	0.44 [0.16, 1.21]	1996
Cremer 1998	44	48	44	48	0.8%	1.00 [0.24, 4.25]	1998
Mehra 1998	137	162	145	167	4.4%	0.83 [0.45, 1.54]	1998
McCarty 2000	188	231	174	211	7.0%	0.93 [0.57, 1.51]	2000
Adam 2000	2531	2900	1198	1352	31.8%	0.88 [0.72, 1.08]	2000
Kaplan 2001	182	198	178	194	3.2%	1.02 [0.50, 2.11]	2001
Norrby 2001	103	138	109	130	4.6%	0.57 [0.31, 1.04]	2001
Portier 2001	76	83	73	78	1.2%	0.74 [0.23, 2.45]	2001
Cohen 2002	233	252	119	130	2.8%	1.13 [0.52, 2.46]	2002
Schaad 2002	131	139	123	130	1.6%	0.93 [0.33, 2.65]	2002
Portier 2002	119	131	122	126	1.3%	0.33 [0.10, 1.04]	2002
Quinn 2003	178	232	170	231	9.0%	1.18 [0.78, 1.80]	2003
Takker 2003	159	170	132	148	2.7%	1.75 [0.79, 3.91]	2003
Kafetzis 2004	77	88	150	177	3.0%	1.26 [0.59, 2.68]	2004
Norrby 2004	339	430	333	428	14.5%	1.06 [0.77, 1.47]	2004
Syrogiannopoulos 2004	344	372	118	124	2.1%	0.62 [0.25, 1.55]	2004
**Total (95% CI)**		**6978**		**4875**	**100.0%**	**0.91 [0.80, 1.04]**	
Total events	6176		4343				

Heterogeneity: Tau² = 0.00; Chi² = 27.81, df = 27 (P = 0.42); I² = 3%

Test for overall effect: Z = 1.37 (P = 0.17)

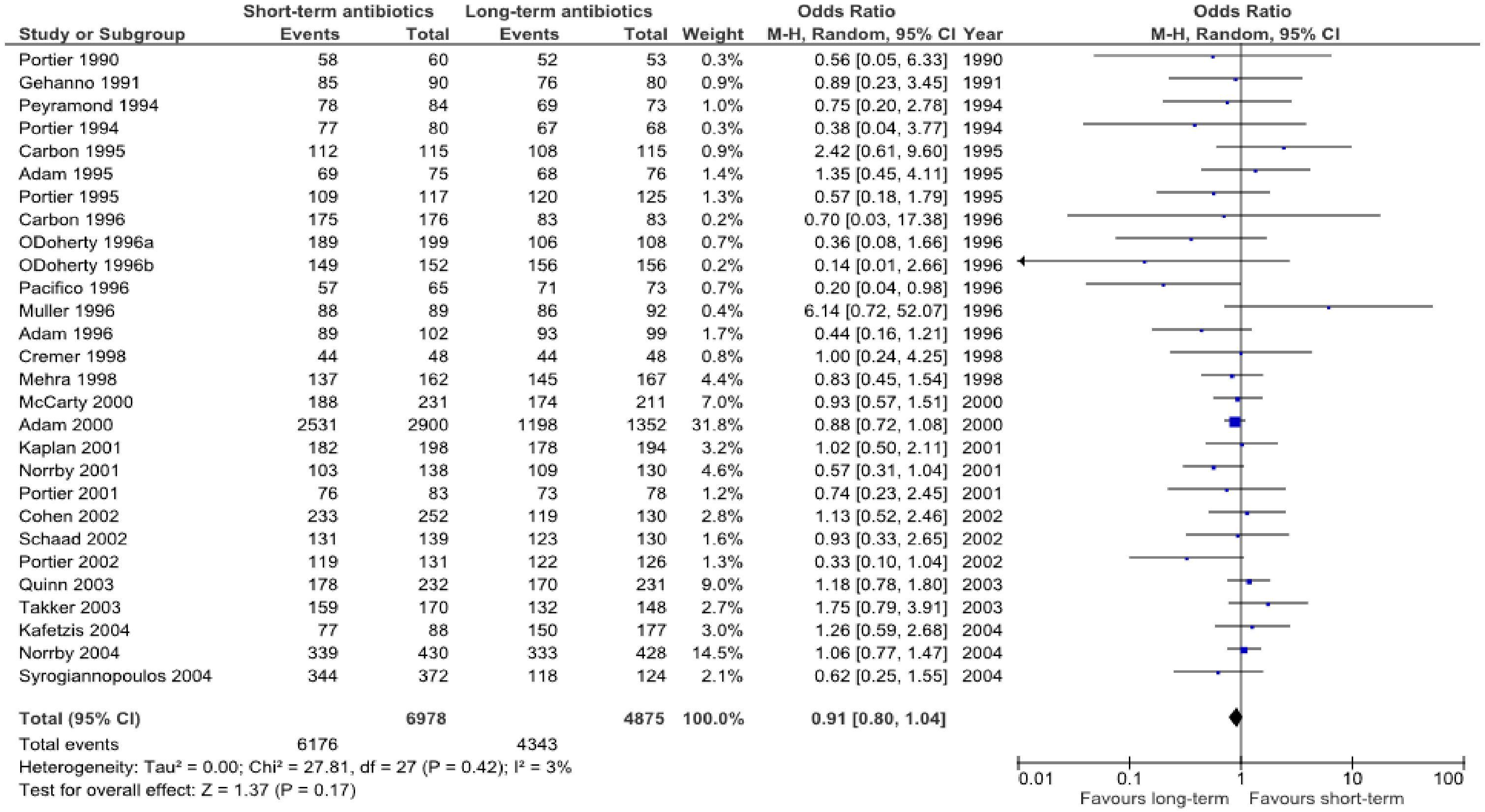

Fig. 7. Late clinical cure of short-term antibiotic therapy compared to long-term regimen treatment

Figure S7. Late clinical cure of short-term antibiotic therapy compared to long-term regimen treatment

Study or Subgroup	Short-term antibiotics Events	Total	Long-term antibiotics Events	Total	Weight	Odds Ratio M-H, Random, 95% CI	Year
Sinanian 1972	22	22	127	132	0.6%	1.94 [0.10, 36.34]	1972
Gerber 1987	59	73	91	99	2.8%	0.37 [0.15, 0.94]	1987
Stromberg 1988	30	66	106	135	3.6%	0.23 [0.12, 0.43]	1988
Portier 1990	51	54	43	48	1.8%	1.98 [0.45, 8.75]	1990
Hooton 1991	123	136	74	84	3.0%	1.28 [0.53, 3.06]	1991
Muller 1993	33	36	33	36	1.5%	1.00 [0.19, 5.32]	1993
Weippl 1993	32	37	34	39	2.0%	0.94 [0.25, 3.56]	1993
Pichichero 1994	113	126	119	151	3.4%	2.34 [1.17, 4.68]	1994
Peyramond 1994	50	54	42	48	2.0%	1.79 [0.47, 6.75]	1994
Portier 1994	67	72	56	61	2.1%	1.20 [0.33, 4.34]	1994
Portier 1995	80	84	78	82	1.9%	1.03 [0.25, 4.25]	1995
Adam 1995	69	75	68	76	2.4%	1.35 [0.45, 4.11]	1995
Schaad 1996	51	93	96	120	3.6%	0.30 [0.17, 0.56]	1996
Pacifico 1996	40	51	67	71	2.2%	0.22 [0.06, 0.73]	1996
ODoherty 1996a	189	199	102	108	2.6%	1.11 [0.39, 3.15]	1996
Adam 1996	77	102	75	99	3.5%	0.99 [0.52, 1.88]	1996
Cremer 1998	19	33	34	39	2.3%	0.20 [0.06, 0.64]	1998
Venuta 1998	60	63	70	74	1.7%	1.14 [0.25, 5.31]	1998
Tack 1998	156	166	152	157	2.5%	0.51 [0.17, 1.54]	1998
McCarty 2000	202	253	198	244	4.0%	0.92 [0.59, 1.43]	2000
Adam 2000	2459	2667	1146	1229	4.4%	0.86 [0.66, 1.11]	2000
Portier 2001	73	83	68	78	2.8%	1.07 [0.42, 2.74]	2001
Norrby 2001	107	138	111	150	3.8%	1.21 [0.71, 2.08]	2001
Esposito 2001	53	56	51	54	1.6%	1.04 [0.20, 5.39]	2001
Kaplan 2001	162	198	176	194	3.6%	0.46 [0.25, 0.84]	2001
Portier 2002	106	126	105	117	3.2%	0.61 [0.28, 1.30]	2002
Schaad 2002	44	141	88	130	3.9%	0.22 [0.13, 0.36]	2002
Cohen 2002	148	196	99	114	3.6%	0.47 [0.25, 0.88]	2002
Quinn 2003	133	187	116	173	4.0%	1.21 [0.77, 1.89]	2003
Takker 2003	131	192	117	168	4.0%	0.94 [0.60, 1.46]	2003
Scholz 2004	406	434	1097	1266	4.1%	2.23 [1.47, 3.39]	2004
Norrby 2004	236	325	225	323	4.3%	1.15 [0.82, 1.62]	2004
Kafetzis 2004	81	88	131	177	3.0%	4.06 [1.75, 9.43]	2004
Syrogiannopoulos 2004	141	243	88	114	3.9%	0.41 [0.25, 0.68]	2004
**Total (95% CI)**		**6869**		**6190**	**100.0%**	**0.81 [0.63, 1.04]**	
Total events	5803		5283				

Heterogeneity: $Tau^2 = 0.36$; $Chi^2 = 141.08$, $df = 33$ ($P < 0.00001$); $I^2 = 77\%$

Test for overall effect: $Z = 1.66$ ($P = 0.10$)

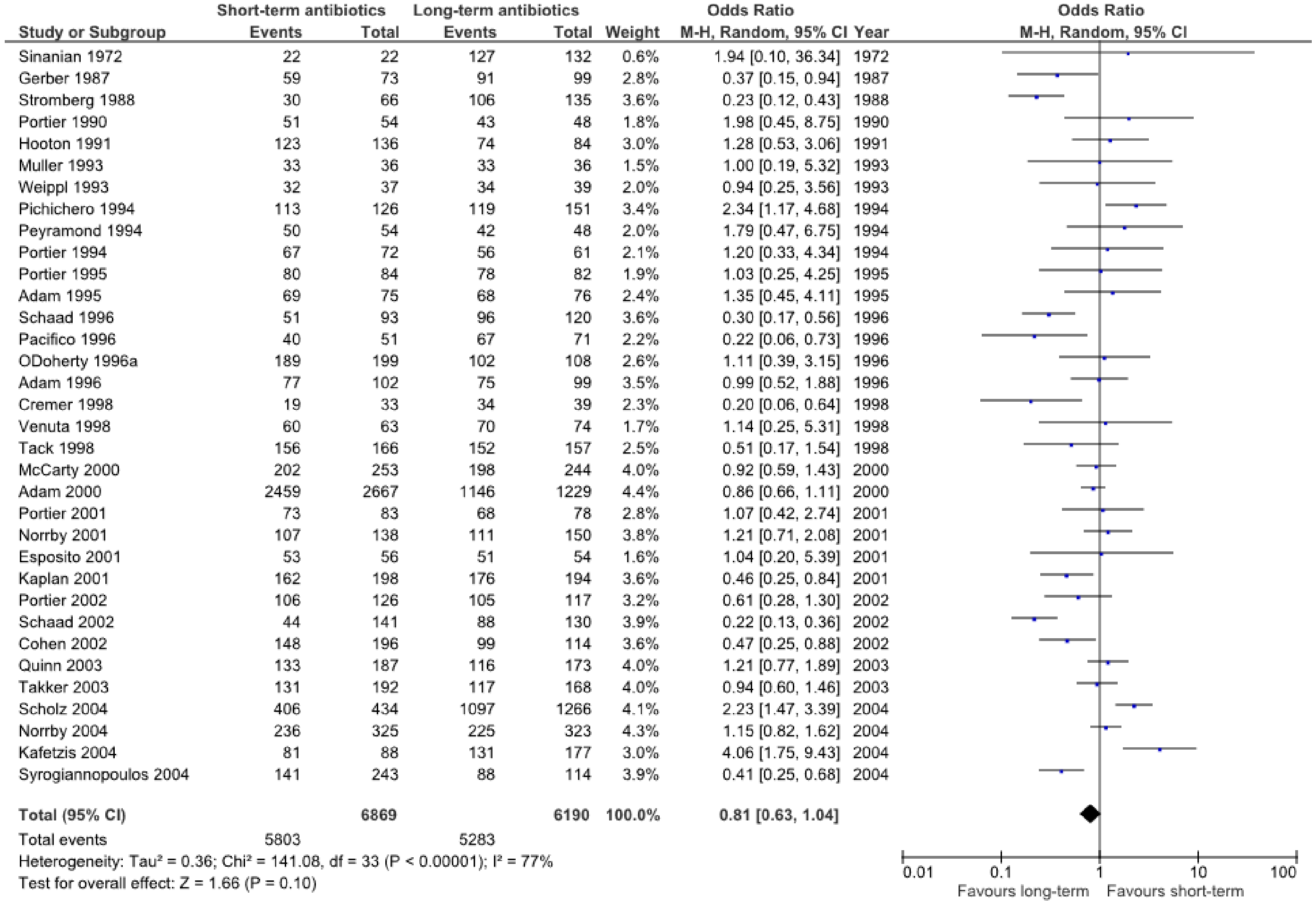

Fig. 8. Late bacteriological eradication of short-term antibiotic therapy compared to long-term regimen treatment

Figure S8. Late bacteriological eradication of short-term antibiotic therapy compared to long-term regimen treatment.

Study or Subgroup	Short-term Events	Short-term Total	Long-term Events	Long-term Total	Weight	Odds Ratio M-H, Random, 95% CI	Year
Sinanian 1972	7	22	17	132	2.3%	3.16 [1.13, 8.86]	1972
Portier 1990	3	65	1	60	0.8%	2.85 [0.29, 28.22]	1990
Hooton 1991	38	229	2	117	1.5%	11.44 [2.71, 48.31]	1991
Gehanno 1991	6	90	8	80	2.1%	0.64 [0.21, 1.94]	1991
Weippl 1993	5	46	8	47	1.9%	0.59 [0.18, 1.97]	1993
Hamill 1993	2	49	0	47	0.5%	5.00 [0.23, 106.95]	1993
Portier 1994	7	165	6	149	2.1%	1.06 [0.35, 3.22]	1994
Pichichero 1994	11	161	18	323	2.9%	1.24 [0.57, 2.70]	1994
Peyramond 1994	9	91	5	0		Not estimable	1994
Carbon 1995	16	119	26	117	3.2%	0.54 [0.27, 1.08]	1995
Adam 1995	5	75	6	76	1.9%	0.83 [0.24, 2.86]	1995
Portier 1995	6	130	2	132	1.3%	3.15 [0.62, 15.88]	1995
Carbon 1996	14	176	13	83	2.8%	0.47 [0.21, 1.04]	1996
Adam 1996	10	115	8	112	2.4%	1.24 [0.47, 3.26]	1996
Schaad 1996	23	164	16	173	3.2%	1.60 [0.81, 3.15]	1996
Pacifico 1996	8	93	5	90	2.0%	1.60 [0.50, 5.09]	1996
ODoherty 1996a	35	326	8	163	2.9%	2.33 [1.06, 5.15]	1996
Tack 1997	30	240	33	242	3.6%	0.90 [0.53, 1.54]	1997
Gendrel 1997	16	149	19	149	3.1%	0.82 [0.41, 1.67]	1997
Venuta 1998	4	74	3	63	1.4%	1.14 [0.25, 5.31]	1998
Tack 1998	51	278	42	280	3.9%	1.27 [0.81, 1.99]	1998
Mehra 1998	15	327	17	324	3.1%	0.87 [0.43, 1.77]	1998
Cremer 1998	11	62	9	60	2.4%	1.22 [0.47, 3.20]	1998
McCarty 2000	35	268	32	260	3.7%	1.07 [0.64, 1.79]	2000
Portier 2001	24	168	16	152	3.2%	1.42 [0.72, 2.78]	2001
Norrby 2001	57	196	41	198	3.8%	1.57 [0.99, 2.49]	2001
Esposito 2001	4	61	6	59	1.7%	0.62 [0.17, 2.32]	2001
Schaad 2002	4	139	6	130	1.8%	0.61 [0.17, 2.22]	2002
Portier 2002	46	177	31	172	3.7%	1.60 [0.96, 2.67]	2002
Cohen 2002	68	333	5	166	2.5%	8.26 [3.26, 20.92]	2002
Takker 2003	61	192	51	168	3.9%	1.07 [0.68, 1.67]	2003
Quinn 2003	154	229	131	228	4.0%	1.52 [1.04, 2.23]	2003
Syrogiannopoulos 2004	69	468	8	158	3.0%	3.24 [1.52, 6.90]	2004
Scholz 2004	13	496	17	1543	3.1%	2.42 [1.17, 5.01]	2004
Norrby 2004	224	427	129	424	4.3%	2.52 [1.91, 3.34]	2004
Kafetzis 2004	4	88	8	177	1.9%	1.01 [0.29, 3.44]	2004
Sakata 2008	3	80	10	151	1.7%	0.55 [0.15, 2.06]	2008
Kuroki 2013	22	47	5	39	2.1%	5.98 [1.99, 17.97]	2013
Skoog Ståhlgren 2019	91	212	125	210	4.0%	0.51 [0.35, 0.75]	2019
**Total (95% CI)**		**6827**		**7254**	**100.0%**	**1.35 [1.08, 1.68]**	
Total events	1211		893				

Heterogeneity: $Tau^2 = 0.27$; $Chi^2 = 119.89$, df = 37 ($P < 0.00001$); $I^2 = 69\%$

Test for overall effect: Z = 2.68 (P = 0.007)

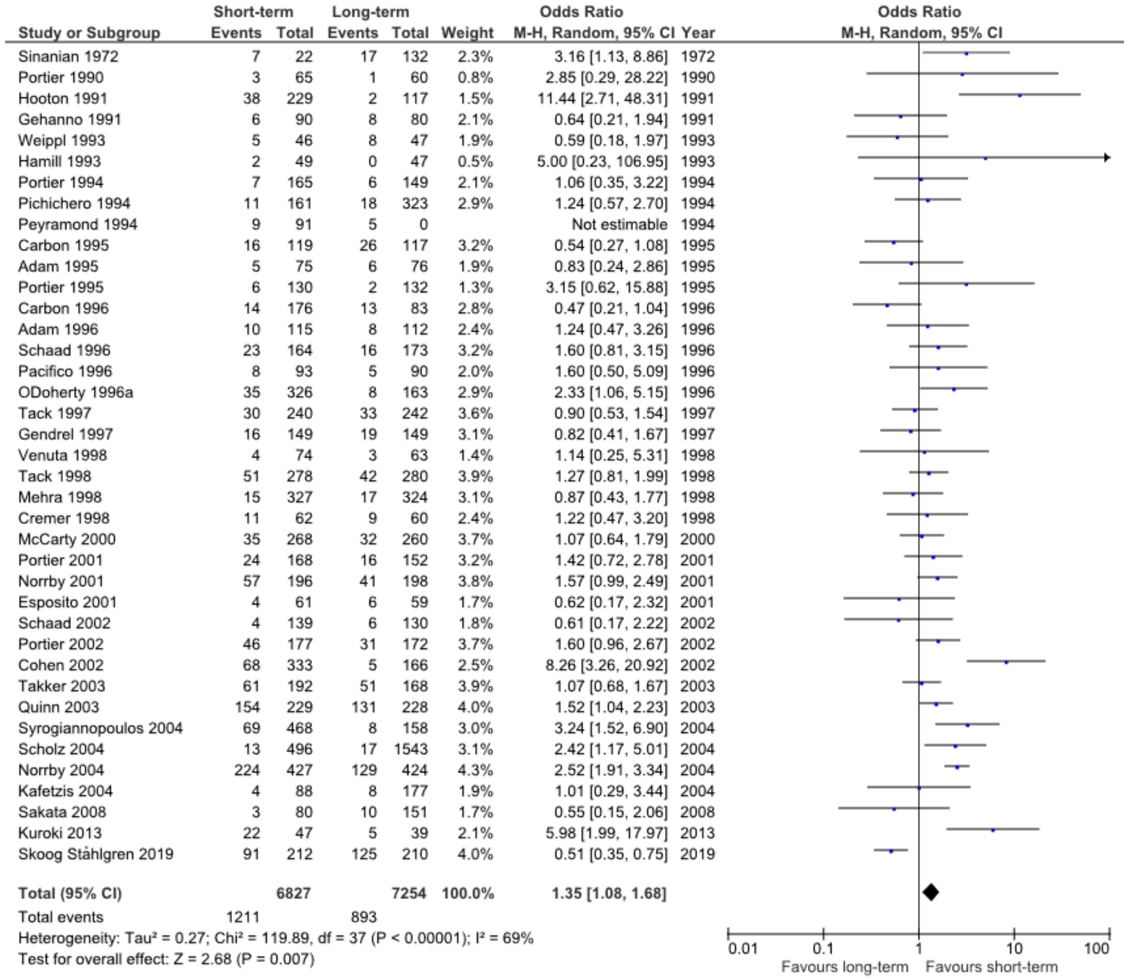

Fig. 9. Adverse effects of short-term antibiotic therapy compared to long-term antibiotic therapy

Figure S9. Adverse effects of short-term antibiotic therapy compared to long-term antibiotic therapy.
